# Use of *Tinospora cordifolia* in HIV infection

**DOI:** 10.4103/0253-7613.62402

**Published:** 2010-02

**Authors:** Shahid Akhtar

**Affiliations:** Department of Pharmacology, Jawaharlal Institute of Postgraduate Medical Education and Research (JIPMER), Pondicherry – 605006, India. E-mail: analyser21@yahoo.co.in

Sir,

With reference to the research article entitled ‘Immunomodulatory effect of *Tinospora cordifolia* extract (TCE) in Human immunodeficiency virus positive patients’[1] published in the May - June 2008 issue of Indian Journal of Pharmacology, there are some issues that need to be clarified by the authors. The baseline parameters of placebo and TCE groups indicate that some patients with CD4 count < 200 / *μ*L were included in the study (289.5 ± 200.3 in TCE group and 282.9 ± 214.1 in placebo group). This is in contrast to the guidelines for initiation of highly active anti retroviral therapy (HAART) in HIV positive patients as laid down by the National AIDS Control Organization (NACO) and the Centre for Disease Control (CDC), Atlanta.[2,3] Advanced HIV infection, as indicated by these low levels of CD4 counts, can rapidly progress to full blown AIDS or encourage opportunistic infections. The use of placebo as control in this trial also goes against the World Medical Association Declaration of Helsinki on Ethical Principles for Medical Research Involving Human Subjects.

Paragraph 29 states –A placebo-controlled trial may be ethically acceptable, even if proven therapy is available, under the following circumstances:

- Where for compelling and scientifically sound methodological reasons its use is necessary to determine the efficacy or safety of a prophylactic, diagnostic or therapeutic method; or

- Where a prophylactic, diagnostic or therapeutic method is being investigated for a minor condition and the patients who receive placebo will not be subject to any additional risk of serious or irreversible harm.

All other provisions of the Declaration of Helsinki must be adhered to, especially the need for appropriate ethical and scientific review.”[4]

In this trial, the patients were placed either on placebo or TCE. A more reasonable approach could have been placing the patients on HAART and using TCE as an add-on therapy.

The authors observe a significant decrease in the eosinophil count in the TCE group, which has been attributed to the immunomodulatory effects of the extract. There was a significant fall in the Hb% in the TCE treatment group. Was it an adverse drug reaction to TCE? The authors have not established the causality in the case of the death of a single patient in the TCE group. The statistical analysis using Kruskal Wallis test is also inappropriate for this kind of data. As compared to the placebo, TCE failed to demonstrate any significant advantage. Yet, at the end of the trial, the patients were offered treatment with TCE instead of being referred to an ART center.
